# miRBase Tracker: keeping track of microRNA annotation changes

**DOI:** 10.1093/database/bau080

**Published:** 2014-08-24

**Authors:** Gert Van Peer, Steve Lefever, Jasper Anckaert, Anneleen Beckers, Ali Rihani, Alan Van Goethem, Pieter-Jan Volders, Fjoralba Zeka, Maté Ongenaert, Pieter Mestdagh, Jo Vandesompele

**Affiliations:** Center for Medical Genetics Ghent, Ghent University, Ghent, Belgium

## Abstract

Since 2002, information on individual microRNAs (miRNAs), such as reference names and sequences, has been stored in miRBase, the reference database for miRNA annotation. As a result of progressive insights into the miRNome and its complexity, miRBase underwent addition and deletion of miRNA records, changes in annotated miRNA sequences and adoption of more complex naming schemes over time. Unfortunately, miRBase does not allow straightforward assessment of these ongoing miRNA annotation changes, which has resulted in substantial ambiguity regarding miRNA identity and sequence in public literature, in target prediction databases and in content on various commercially available analytical platforms. As a result, correct interpretation, comparison and integration of miRNA study results are compromised, which we demonstrate here by assessing the impact of ignoring sequence annotation changes. To address this problem, we developed miRBase Tracker (www.mirbasetracker.org), an easy-to-use online database that keeps track of all historical and current miRNA annotation present in the miRBase database. Three basic functionalities allow researchers to keep their miRNA annotation up-to-date, reannotate analytical miRNA platforms and link published results with outdated annotation to the latest miRBase release. We expect miRBase Tracker to increase the transparency and annotation accuracy in the field of miRNA research.

**Database URL:** www.mirbasetracker.org

## Introduction

MicroRNAs (miRNAs) represent the most widely studied class of noncoding RNA molecules. Since their discovery in 1993, the number of scientific reports on miRNAs has increased exponentially (1) and a role as crucial gene expression regulators involved in numerous aspects of both the normal and the diseased cell's physiology has been demonstrated in many organisms. In the canonical miRNA pathway, mature miRNAs are processed from hairpin precursors and guide an effector protein complex, named miRISC, to regions of partial or complete complementarity in target mRNA molecules. miRISC complexes subsequently exert the miRNA-mediated repression by inducing destabilization, degradation or translational inhibition of target molecules (2).

The pivotal role of miRNAs in regulating gene expression is reflected by various achievements in biomedical research and human medicine, and the impact they are starting to have on patient management. Cases in point are the development of diagnostic and prognostic miRNA expression signatures for various human pathologies (3), and the prospect of miRNA-based therapeutics for viral diseases and cancer (3), with hsa-miR-34a-5p replacement therapy in liver cancer patients entering clinical trials (4) and antisense agents directed against hsa-miR-122-5p at the doorstep of market approval for treating hepatitis C infection (5).

### The miRBase database is highly dynamic

With the development of the miRBase database, the Wellcome Trust Sanger Institute initiated an effort to unambiguously annotate miRNAs and to enable unified use of miRNA sequences throughout the research community (1, 6, 7). The miRBase database, originally the miRNA registry, has been the reference database for miRNA annotation since 2002 (8). It provides a central place for collecting all known precursor and mature miRNA sequences, along with primary evidence for their existence. In addition, miRBase has put forward the miRNA nomenclature conventions that have been accepted by the research community.

**Figure 1.**
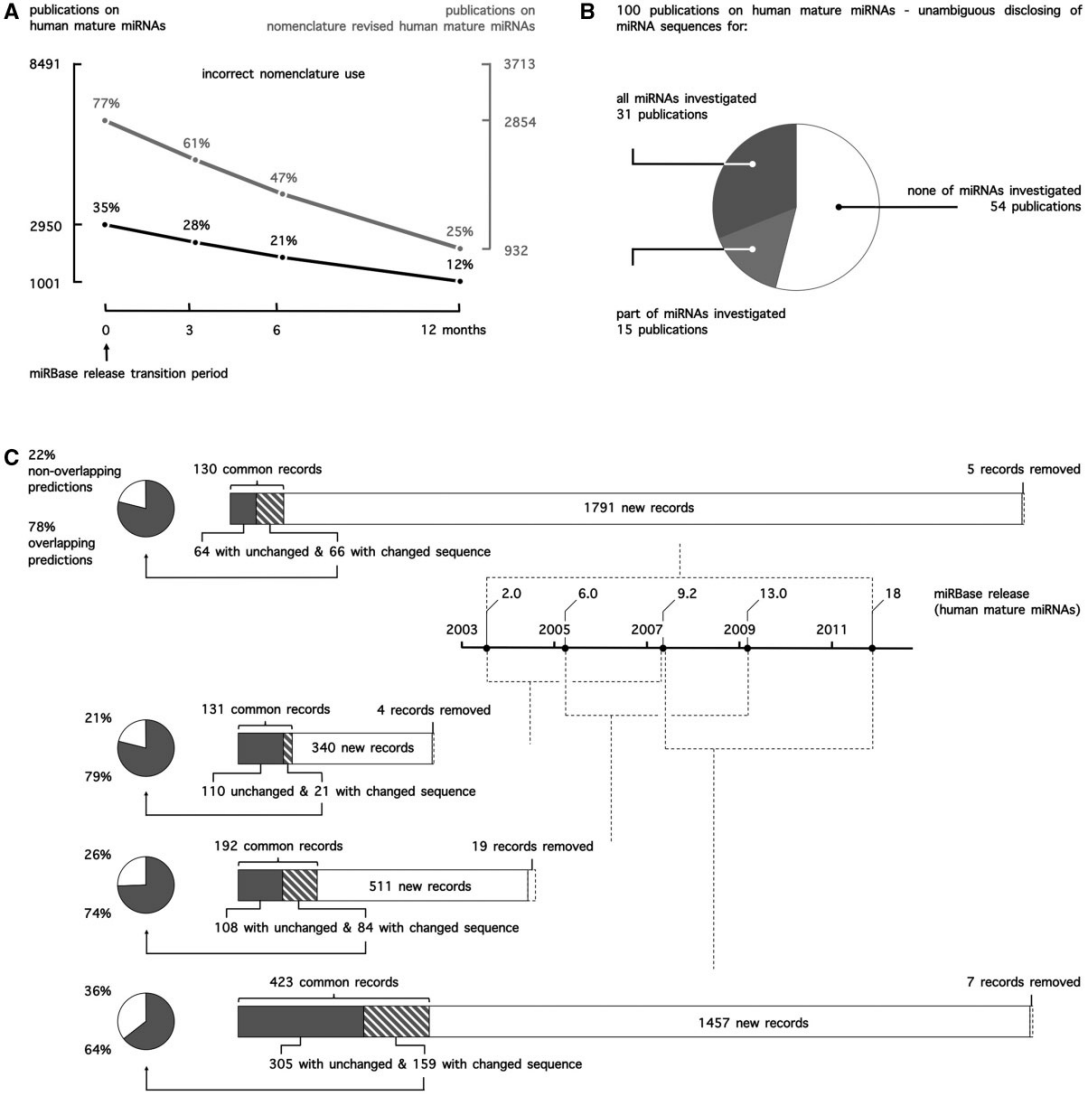
(**A**) miRNA nomenclature use in public literature. Publications on human mature miRNAs in PubMed were curated for correct use of nomenclature. To account for potential incorrect use because of the release of one or multiple miRBase versions during peer review, any of the applicable conventions up until 12 months before publication (miRBase release transition period) were accepted. (**B**) miRNA sequence reporting in public literature. 100 publications on human mature miRNAs were curated for the unambiguous disclosing of miRNA sequences. Accepted reporting was the mature miRNA sequence itself (e.g. ACAAGUCAGGCUCUUGGGACCU), the miRNA name in combination with the miRBase release (e.g. rno-miR-125b-2-3p, miRBase 21) or the miRNA accession number in combination with the miRBase release (e.g. MIMAT0026467, miRBase 21). (**C**) Evolution of miRBase for human mature miRNA records and the impact of miRNA sequence annotation changes on target prediction.

Mainly as a result of the increasing number of small RNA sequencing efforts, the database has grown exponentially since its initial publication (Figure 1C) (1), going from 218 precursor and 218 mature miRNAs from five species annotated in the first release (miRBase release 1.0, December 2002) to 28 645 precursor and 35 828 mature miRNAs from 223 species annotated in the release at time of publication (miRBase release 21, June 2014). In addition, technical advances in the field of massively parallel sequencing, with increasing coverage depths, have not only led to the detection of miRNAs of ever lower abundance but also to more reliable sequence annotation.

These evolutions have resulted in an organically grown database with the number of records as well as their respective contents changing over time. Unfortunately, in its current database structure, miRBase does not allow straightforward assessment of these annotation changes, resulting in ambiguous and erroneous application of miRNA nomenclature and sequence information in the research community. With miRBase Tracker, we provide a tool to resolve this ambiguity.

## Results

### Changing nomenclature creates confusion about miRNA identity in public literature

‘What's in a name?’ Shakespeare once wrote. For miRNAs this certainly holds true. Using only a miRNA's name to reference it seems natural, but it comes with various limitations. For one, a miRNA name does not unambiguously identify a miRNA sequence. A miRNA sequence is under constant revision and can change as new (more accurate) data becomes available. In particular cases, the same name has also been used for different miRNA sequences, as is for instance the case for the widely studied human mature hsa-miR-34b (9, 10). For another, miRNA names constantly evolve, even while remaining associated with the same molecule. The gradual uncovering of the miRNome's complexity, with identification of homologous miRNA loci, miRNA families and the awareness that both strands of the precursor stem can produce functional miRNAs, has required more complex naming schemes, resulting in changing nomenclature conventions. The miRBase database has tried to convey this increasing biological complexity by adding different suffixes to precursor and mature miRNA names.

As researchers typically rely only on names to reference miRNAs in their publications, this evolution of nomenclature has created a lot of ambiguity, and it is often difficult to unambiguously identify the miRNA molecule under study. Linking different publications, target prediction database entries and commercial research products has proven to be more deceptive than anticipated, especially when sequence information is unavailable. This ambiguity is apparent from the observation that 12% of publications (Figure 1A) on human mature miRNAs use outdated or incorrect miRNA names, when referencing to nomenclature conventions applicable at the time of publication. When focusing on publications on miRNAs whose name has been revised at least once at time of publication (44% of publications), this number even rises to 25%.

Using outdated or incorrect nomenclature hints at an unawareness of annotation updates or an inability to keep up with them; it does not imply erroneous research conclusions per se. The resulting miRNA identity ambiguities, however, at least hamper correct interpretation and comparison of independent studies and thus indirectly affect research quality. Even when a miRNA name can unambiguously be linked to a miRNA molecule, caution is still warranted, as annotated miRNA sequences are not stable, but can subtly change.

### Ignoring changes in sequence annotation impacts research outcome

Altered miRNA sequence annotation can be a consequence of improved sequencing accuracy, but can also reflect true biological variability. Subtle sequence variants can arise from homologous miRNA loci or by differential processing of the product of a single miRNA locus, which are then called isomiRs (11). Irrespective of its underlying cause, it is important for researches to be aware of these changing sequence annotations.

A miRNA's sequence and function are closely linked. Even a small sequence variation can have a profound impact on targeting specificity, especially when the new sequence comes with a different seed region. Single nucleotide polymorphisms and point mutations in mature miRNAs have been shown to divert them to other mRNA targets (12). Furthermore, numerous examples exist of single nucleotide changes in 3′UTR regions that either create or disrupt a functional binding site (12). A striking example of the tight relationship between sequence and function is observed in high-grade glioma patients, where lowered adenosine-to-inosine editing of a single nucleotide in miR-376a-5p leads to loss of AMFR protein repression and increased inhibition of RAP2A, which contributes to a more aggressive disease and correlates with reduced patient survival (13).

Although it is desirable to report on up-to-date miRNA sequences, the use of outdated sequences in itself is not problematic. After all, publications do not suddenly lose their value upon a miRBase update. Furthermore, it is hard to claim previously annotated sequences to be erroneous, with annotation continuously changing, sometimes back and forth, and with the notion that different versions of an annotated miRNA sequence might just represent isomiRs. The problem is founded in ignoring these sequence annotation differences, when comparing studies that actually investigated slightly different sequences.

A systematic and in-depth assessment of the impact of ignoring miRNA sequence annotation changes on research conclusions in literature is difficult. As a surrogate, we assessed how sequence annotation changes affect miRNA target prediction. Measuring this impact is a relevant alternative, as target prediction is generally one of the first steps undertaken towards obtaining a clue on a given miRNA’s function. We performed and compared human target predictions for five different miRBase releases (Figure 1C) and found between 21 and 36% of nonoverlapping predictions between any two releases because of sequence changes alone (not taking into account differential predictions due to newly added or deleted miRNA records), clearly demonstrating that ignoring sequence annotation changes drastically impacts functional miRNA research.

Of note, not all online miRNA target prediction tools and databases are keeping their miRNA sequence annotation up-to-date. PicTar (14) predictions, for instance, are still based on the miRBase release 5.0 annotation (2004). Since then, about half of the human mature miRNA sequences annotated in that release (92 of 189) have changed, 2402 new miRNAs have been added to the database and 3 miRNA records have been deleted (compared with release 21). Despite being dramatically outdated, PicTar still remains one of the most widely used algorithms in the field, with 270 citations in 2013 alone and probably much more unreferenced use, underscoring many researchers’ unawareness of the problem.

### Accession numbers do not solve the problem of miRNA annotation dynamics

In principle, annotation changes can be overcome by providing each miRNA specimen with a unique and stable identifier or accession number, analogous to for instance the RefSeq system for nucleotide sequence and protein annotation (15). Accession numbers for precursor miRNAs were introduced in the first miRBase release (2002; e.g. MI0000116 for dme-mir-1), whereas mature miRNA accession numbers were only introduced in release 6.0 (2005; e.g. MIMAT0000896 for rno-miR-292-5p). Evidently, as the information linked to a miRNA record is prone to change, this identifier only holds unique information when accompanied by a version number, again much like the situation for RefSeq records. Unfortunately, the miRBase accession number system does not include version numbers. Therefore, unambiguous reporting of a miRNA can only be achieved by either disclosing the sequence or, alternatively, providing its name or accession number explicitly in combination with the release version of the miRBase database. Considering the impact of sequence changes on miRNA function and research outcome, it is worrisome that 69 of 100 manually curated articles do not unambiguously report sequences of all investigated miRNAs (Figure 1B).

### miRBase Tracker allows easy tracking of miRNA annotation changes

As demonstrated above, the organically grown miRBase has lead to substantial risks of miRNA identity confusion and ignoring sequence annotation changes. To help researchers keep track of the annotation of their miRNAs of interest, we developed miRBase Tracker, an easy-to-use online database and webtool (www.mirbasetracker.org) that stores all miRBase releases in a logically organized MySQL database. Three useful query options are presented to the user.

**Figure 2.**
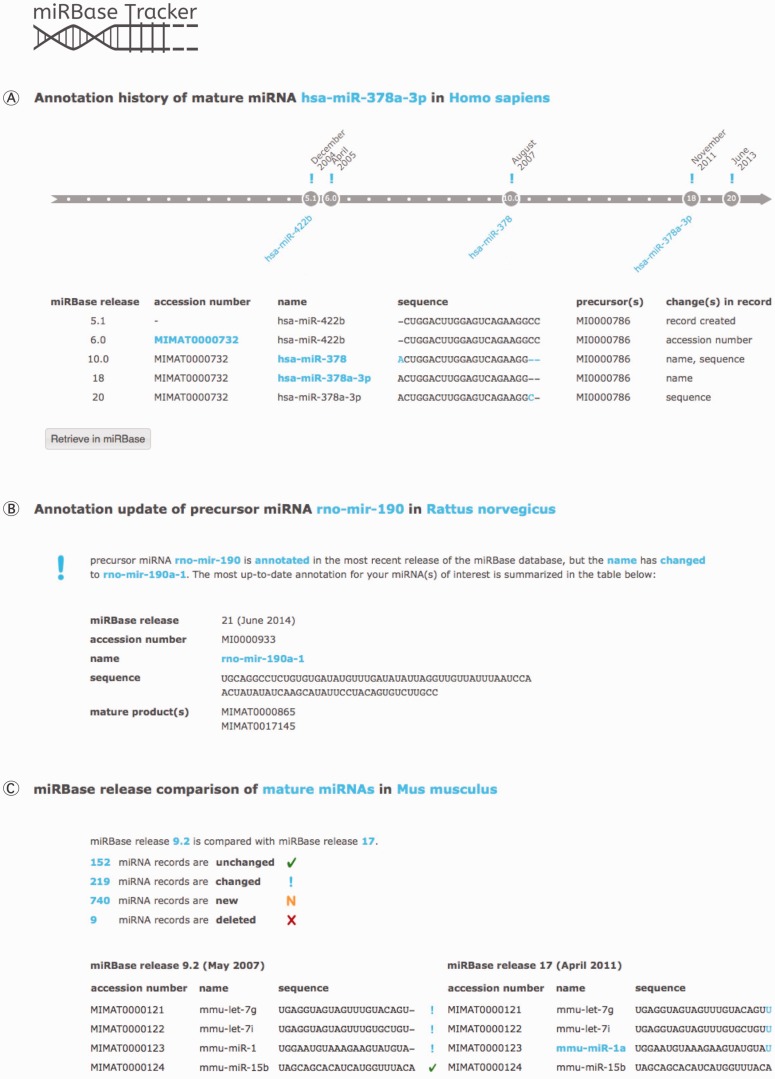
miRBase Tracker functionalities. (**A**) *miRNA history* query: annotation history of the human mature miRNA ‘hsa-miR-378a-3p’. (**B**) *miRNA update* query: updated annotation (miRBase release 21 at time of publication) of the rat precursor miRNA ‘rno-mir-190’. (**C**) *miRBase release comparison* query: comparison of miRBase release 9.2 with 17 for mouse mature miRNAs.

With a *miRNA history* query (Figure 2A), the user gets an overview of the annotation changes of any mature or precursor miRNA of interest throughout the different miRBase releases. Searching for a miRNA name, sequence or accession number will show the evolution of all nomenclature, sequence information and other annotation linked to it over time.

Retrieving the most up-to-date annotation for any precursor or mature miRNA name, sequence or accession number annotated in any miRBase release can be achieved by performing a *miRNA update* query (Figure 2B). If needed, batch files containing information on multiple miRNAs can be uploaded, facilitating reannotation of analytical miRNA platforms and of outdated miRNA gene lists in literature.

The *miRBase release comparison* query option (Figure 2C) compares any two miRBase releases for both mature and precursor miRNAs within any species. Users get an overview showing which miRNA records were added, removed, changed or remained unchanged, along with summarizing statistics.

## Discussion

The information stored in the miRBase reference database for miRNA annotation is highly dynamic. New records are added on discovery of new miRNA entities, existing records are deleted if there is no longer sufficient evidence for existence of the miRNAs covered and redundant records covering the same miRNA are merged. Sequences get revised as additional data become available and nomenclature changes in an effort to convey as much biological complexity through miRNA naming schemes as possible. Because the miRBase database structure does not allow straightforward appreciation of these continuous annotation changes, substantial ambiguity has arisen in the field of miRNA research, with confusion about miRNA (sequence) identity in public literature, in target prediction databases and on analytical platforms. Companies selling commercial miRNA research products have further complicated the situation, as they often apply their own nomenclature, fail to update product annotation or recycle product IDs, creating a false sense of annotation stability. Researchers also contribute to the problem, as they fail to unambiguously report the miRNAs they investigate, making it impossible to reliably build on their studies. Therefore, we would like to encourage miRNA researchers to no longer solely (and conveniently) rely on miRNA names, but to also disclose the sequences or, alternatively, provide the miRNA names or accession numbers explicitly in combination with the miRBase release version.

Conclusions reported in miRNA literature have most likely suffered from this ambiguity. Although difficult to assess, miRNA identity confusion may have led to unjustified comparison and integration of results from studies investigating unrelated miRNA sequences. In addition, in some instances, comparison of study results on (supposedly) identical miRNA molecules has inevitably ignored subtle sequence annotation changes. As we demonstrate here, subtle sequence annotation changes can dramatically impact study conclusions, as on average one-third of the targetome is expected to change for miRNAs undergoing a sequence update. Unfortunately, the poor unambiguous sequence reporting in literature, together with the widespread use of online target prediction databases relying on outdated annotation, point at an unawareness of the problem in the field. Keeping track of correct miRNA annotation and aligning annotation of different data sources should, however, be considered imperative to high-quality miRNA research. Although possibly challenging when integrating high-throughput and/or whole-genome miRNA data sets, at least when prioritizing miRNAs for further investigation, it becomes the researcher's responsibility to retrospectively verify correct alignment of annotation. Furthermore, one must always strive to respect the most up-to-date annotation, as it represents the most accurate view on the miRNome at any given time.

miRBase Tracker is a framework on top of miRBase that, in addition to providing miRBase's basic annotation information, allows researchers to keep track of miRNA annotation changes and facilitates reannotation. In the future, keeping track of correct miRNA annotation is speculated to become even more challenging owing to the discovery of isomiRs (11), subtle sequence variants arising by differential processing of precursor and mature miRNAs depending on tissue type, developmental stage and physiological conditions. At present, miRBase ignores these bona fide variants and miRNAs are annotated as single canonical sequences, representing for each miRNA the predominant isomiR as known to date. Respecting biological reality, however, would require miRBase to couple multiple sequence variants to a single miRNA record. A context-dependent pursuit of particular isomiRs for individual miRNAs will then become the next challenge for the miRNA researcher. At present, the predominant isomiRs present in miRBase-curated data sets can change, which—in addition to improved sequencing accuracy—probably also partly explains the dynamic in sequence annotation.

Given the prospect of increasing complexity in absence of a transparent database that allows straightforward assessment of miRNA annotation changes, we believe that miRBase Tracker will be crucial in overcoming the growing ambiguity in the field of miRNA research.

## Methods

### Nomenclature use in public literature

miRNA nomenclature usage in public literature was assessed by means of an automated text-mining approach similar to the one used for the PubMeth database (16). Briefly, NCBI's PubMed database was queried with every human mature miRNA name—and its textual variants—that was ever annotated in the miRBase database (up until release 19), in combination with the MeSH term ‘human’. 8491 publications were retrieved over a 16-year publication time frame (March 1997–March 2013) (Supplementary Table S1). miRNA nomenclature used in each publication was then referenced to nomenclature conventions applicable at the time of publication and in the preceding year.

### Sequence reporting in public literature

miRNA sequence reporting in public literature was manually assessed. 100 publications on human mature miRNAs were randomly selected over a 3-year publication time frame (1 July 2010–30 June 2013) (Supplementary Table S2). Article full-texts, figures and supplementary files were checked for unambiguous reporting of the sequence(s) of the investigated miRNA(s). Accepted reporting was either the mature sequence itself or, alternatively, the name or accession number in combination with a miRBase release version.

### Differential target prediction

The MirTarget2 (17) algorithm was used to predict 3′UTR targets (RefSeq database release 54) of all human mature miRNA sequences annotated in five relatively equally spaced releases of the miRBase database (2.0, 6.0, 9.2, 13.0 and 18). To assess the degree of differential prediction exclusively attributable to sequence annotation changes, predictions from newly added or deleted miRNA records were not considered, and only predictions from records common to both releases but having a sequence change were included in the analysis. For any combination of two releases, prediction overlap was calculated as the ratio of the number of common interactions to the total number of unique interactions predicted using both releases.

### Database structure and content

miRBase annotation files miRNA.dat (published miRNA data in EMBL format), mature.fa (mature miRNA sequences in FASTA format) and miRNA.dead (entries removed from the database) are retrieved from the miRBase FTP server (ftp://mirbase.org) for all miRBase releases. Files are locally parsed to extract relevant annotation for mature and precursor miRNAs (accession number, name, sequence) and to determine relationships between precursor and mature miRNA records (precursor parents, mature products) in each release. Record annotation is compared between subsequent releases, using accession numbers as the unique identifier for miRNA records. As accession numbers for mature miRNAs were introduced only in release 6.0, files up to miRBase release 5.1 were manually curated to match (later) accession numbers. For each annotation change, an entry is created in a MySQL table containing the type of change, the release of occurrence and a change event identifier, linking to a second table holding the annotation for the miRNA record after the change. The presented database allows quick querying of the annotation history of any miRNA record and is computationally nonintensive. The database can be queried online at www.mirbasetracker.org.

## Supplementary Data

Supplementary data are available at *Database* online.

Supplementary Data
