# Supplementary material for: miRBase Tracker: keeping track of microRNA annotation changes
Source: Database (Oxford). 2014 Aug 24;2014:bau080. doi: 10.1093/database/bau080 (PMC4142392; doi:10.1093/database/bau080)
Supplement: Supplementary Data [file supp_2014_bau080_index.html]

miRBase Tracker: keeping track of microRNA annotation changes — Supplementary Data 

# miRBase Tracker: keeping track of microRNA annotation changes

## Supplementary Data

files

**Files in this Data Supplement:**

- Supplementary Data - xlsx file
- Supplementary Data - xlsx file
